# Attractor detection and enumeration algorithms for Boolean networks

**DOI:** 10.1016/j.csbj.2022.05.027

**Published:** 2022-05-21

**Authors:** Tomoya Mori, Tatsuya Akutsu

**Affiliations:** Bioinformatics Center, Institute for Chemical Research, Kyoto University, Kyoto 611-0011, Japan

**Keywords:** Boolean network, Singleton attractor, Periodic attractor, Computational complexity, SAT, Nested canalyzing function

## Abstract

The Boolean network (BN) is a mathematical model used to represent various biological processes such as gene regulatory networks. The state of a BN is determined from the previous state and eventually reaches a stable state called an attractor. Due to its significance for elucidating the whole system, extensive studies have been conducted on analysis of attractors. However, the problem of detecting an attractor from a given BN has been shown to be NP-hard, and for general BNs, the time complexity of most existing algorithms is not guaranteed to be less than $O(2^{n})$. Therefore, the computational difficulty of attractor detection has been a big obstacle for analysis of BNs. This review highlights singleton/periodic attractor detection algorithms that have guaranteed computational complexities less than $O(2^{n})$ time for particular classes of BNs under synchronous update in which the maximum indegree is limited to a constant, each Boolean function is AND or OR of literals, or each Boolean function is given as a nested canalyzing function. We also briefly review practically efficient algorithms for the problem.

## 1 Introduction

The *Boolean network* (BN) [1], [2], [3] is a mathematical model that is used to represent various biological processes such as gene regulatory networks [4], [5], [6], neural networks [7], cell cycle control networks [8], and signal transduction networks [9], [10], [11], [12]. For example, when modeling a gene regulatory network, each node corresponds to a gene and is assigned a Boolean value of 0 (FALSE) or 1 (TRUE), which means the gene is inactive or active, respectively. The state of each node is updated according to a Boolean function assigned to it, i.e., the edges between nodes correspond to the regulation relationships between the nodes. In addition, the state of the entire system is called a *global state*, which eventually reaches one of two types of stable states: *singleton attractor* (*point attractor*) or *periodic attractor* (*cyclic attractor*) by repeating the update of the state. Once the global state reaches a singleton attractor, it will always remain in the same state, whereas a periodic attractor consists of multiple global states that are cyclically visited. There is a close relationship between attractors and biological processes. For example, in the case of a gene regulatory network, an attractor is a stable state of the entire system and is considered to be a stable state of a cell. Therefore, one attractor is often regarded as one cell type [3], [13], [14], and extensive studies have been conducted on attractor detection, due to its significance for elucidating the system. Although it might be pointed out that a BN is a fairly simplified model and it deviates from the actual biological systems, it is meaningful to start the analysis with a simple model in order to decipher a complex system.

A BN of *n* nodes has $2^{n}$ global states. Thus, if we want to detect or enumerate the attractors of a given BN, we can simply update all global states as initial states and find out which initial state reaches which stable state. If *n* is sufficiently small, this approach is not problematic. When *n* is large, however, a large amount of computational resources is required. Therefore, algorithms for biological BNs have so far been limited to small networks in most cases [15]. Furthermore, from a theoretical view point, detection of a singleton attractor and enumeration of singleton attractors have been shown to be NP-hard [16], [17] and #P-hard [16], respectively. In fact, there is no existing method that works in $o(2^{n})$ time (i.e., provably faster than in $O(2^{n})$ time) for general BNs. Therefore, algorithms that work efficiently for limited classes of BNs have been proposed [18], [19]. For instance, there are some algorithms that detect a singleton attractor in polynomial time for very restricted classes of BNs [20], [21] and several $o(2^{n})$ time algorithms for reasonably wide classes of BNs [6]. On the other hand, no $o(2^{n})$ time algorithm is known for detection of a periodic attractor of period 3 or more for a reasonably wide class of BNs. This may be because detection of an attractor with a long period has been suggested to be PSPACE-hard [6], which is more difficult than problems belonging to class NP unless NP = PSPACE. This theoretical observation also suggests a practical difficulty of detection of a long periodic attractor because PSPACE-hard problems may not be efficiently solved by using practical solvers for NP-hard problems (e.g., SAT-solver, ILP-solver).

In this paper, we review $o(2^{n})$ time algorithms for singleton/periodic attractor detection for particular classes of BNs under synchronous update in which the maximum indegree is limited to a constant, each Boolean function is AND or OR of literals, or each Boolean function is given as a nested canalyzing function. The reasons why the time complexities of these special classes of BNs have been well-discussed and we focus on them in this paper are (i) each Boolean function can be represented and evaluated in constant space and constant time by limiting the maximum indegree, (ii) it is possible to develop $o(2^{n})$ time algorithms by introducing some constraints on types of Boolean functions even when there is no constraint on the maximum indgree, (iii) biologically important functions often have a nested canalyzing form [22], [23], [24], [25], [26], and (iv) there are some other BN classes (e.g., *monotonic* BNs [27], [28], [29], *monomials* BNs [30], [31], *semilattice* BNs [32], BNs whose functions consist of only XOR operations [33], BNs whose functions consist of only AND and NOT operations [34]) but algorithms guaranteed to work in $o(2^{n})$ time for the classes have not been proposed as far as we know. We also briefly review practical algorithms for detection and enumeration of attractors for BNs.

## 2 Boolean network and attractors

**Fig. 1:**
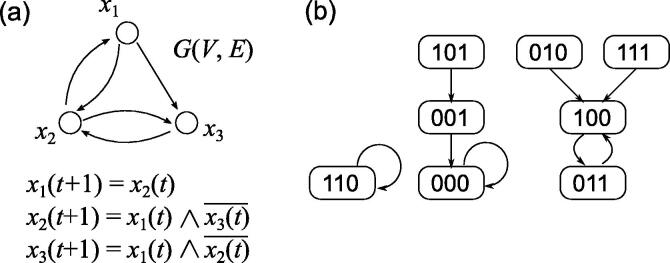
Example of (a) Boolean network and (b) its state transition diagram.

A BN N(V,F) consists of a set of nodes $V=\{x_{1},x_{2},\ldots ,x_{n}\}$ and a set of Boolean functions $F=\{f_{1},f_{2},\ldots ,f_{n}\}$. Each $x_{i}$ takes a value of 0 (FALSE) or 1 (TRUE), and in the case of a gene regulatory network, $x_{i}$ corresponds to the gene *i*, and 0 and 1 indicate that the gene is inactive or active, respectively. In particular, when writing $x_{i}(t)$, it indicates the state of the gene *i* at time *t*, and the *global state* of the entire system at time *t* is denoted by $x(t)=[x_{1}(t),x_{2}(t),\ldots ,x_{n}(t)]$. A Boolean function $f_{i}(x_{i_{1}},x_{i_{2}},\ldots ,x_{i_{k}})$ is assigned to each $x_{i}$ and it indicates a rule that $x_{i}$ is controlled by specific input nodes $x_{i_{1}},x_{i_{2}},\ldots ,x_{i_{k}}$. Here, let $\mathrm{IN}(x_{i})=\{x_{i_{1}},x_{i_{2}},\ldots ,x_{i_{k}}\}$ be a set of input nodes for $x_{i}$. The number of input nodes is denoted by $\left|\mathrm{IN}(x_{i})\right|$ and is called *indegree* of $x_{i}$. We use *K* to denote the maximum indegree for a BN. The state of $x_{i}$ at time t+1 is determined from the state of $\mathrm{IN}(x_{i})$ at time *t* according to the corresponding Boolean function $x_{i}(t+1)=f_{i}(x_{i_{1}}(t),x_{i_{2}}(t),\ldots ,x_{i_{k}}(t))$. This may be simplified and written as $x_{i}(t+1)=f_{i}(x_{1}(t),x_{2}(t),\ldots ,x_{n}(t))$ if there is no confusion. Furthermore, the global state at time t+1 can be written as x(t+1)=f(x(t)). The network structure of BN is represented by a directed graph G(V,E), where $E=\{(x_{i_{j}},x_{i})|x_{i_{j}}\in \mathrm{IN}(x_{i})\}$. As an example, Fig. 1(a) shows the following BN and the corresponding graph:

$$\begin{array}{cc} x_{1}(t+1)= & x_{2}(t),\\ x_{2}(t+1)= & x_{1}(t)\wedge \overset{‾}{x_{3}(t)},\\ x_{3}(t+1)= & x_{1}(t)\wedge \overset{‾}{x_{2}(t)}, \end{array}$$

where x∧y and $\overset{‾}{x}$ denote conjunction (AND) of *x* and *y*, and negation (NOT) of *x*, respectively. Since this BN has three nodes, the number of global states is $2^{3}$ in total. At each time step, the transition of each global state is computed by x(t+1)=f(x(t)) and a directed graph representing such transition of the states is called a *state transition diagram* (Fig. 1(b)). As we can see from this state transition diagram, the global state at time t+1 is uniquely determined by the global state at time *t*. For example, if x(t)=[1,0,1], the global state will be [0,0,1] at time t+1 and then [0,0,0] at time t+2. However, once it reaches [0,0,0], it remains [0,0,0] no matter how many updates are repeated. This state is called a *singleton attractor* or *point attractor*. Meanwhile, the states [1,0,0] and [0,1,1] repeat the transition between these states and are called a *periodic attractor* or *cyclic attractor*. More generally, an attractor is represented by a set of states $\left\{a_{0},a_{1},\ldots ,a_{p-1}\right\}$, where $a_{i+1}=f(a_{i})$ (i=0,1,…,p-2) and $a_{0}=f(a_{p-1})$ hold. If p=1, it corresponds to a singleton attractor. If p>1, it corresponds to a periodic attractor and *p* is called the *period* of the attractor (the more detailed reviews on the attractor can be found in [35]). Since an attractor can be regarded as a stable state of the entire system, detection and enumeration of attractors are important analysis for detailed understanding of the system. Therefore, extensive studies have been done on the distribution and length of attractors. In particular, the *NK model* of BN is often focused on, which consists of *N* nodes and each node has *K* input nodes randomly selected and is assigned a Boolean function randomly selected from all possible $2^{2^{K}}$ functions. In the analysis of this NK model, it is well known that the expected number of singleton attractors is 1 for every K>0 and n⩾K [36], [37]. As for the length and distribution of periodic attractors in the NK model, several theoretical studies have been done [38], [39].

There exist mainly two types of BNs, *synchronous BNs* whose states of all nodes are updated all at once and *asynchronous BNs* whose states of nodes are updated asynchronously such as *random order asynchronous*, *general asynchronous*, and *deterministic asynchronous* update models [11]. Asynchronous BNs are often employed since the update strategy is reasonable to take into account biological processes which occur in different time scales [6], [40]. However, each state of the state transition diagram of an asynchronous BN may have more than one outgoing edges. Therefore, an attractor in an asynchronous BN is often defined as a *strong connected component* (SCC) without outgoing edges (referred to as *bottom SCC* or *terminal SCC*), and the attractor detection is considered as the bottom SCC detection [41]. Such a complex structure of attractor prevents us from applying SAT-based algorithm (described in Section 3.1.1) efficiently since each SAT formula becomes large [42]. Meanwhile, the fact that the synchronous model and the general asynchronous model have the same set of singleton attractors can be shown [6]. Although we deal with synchronous BNs in this paper unless otherwise stated since extensive studies on attractor detection have been done due to their simplicity, some practical attractor detection algorithms for asynchronous BNs are described in Section 5. In addition to synchronous and asynchronous BNs, another semantics on BNs called *most permissive* has been proposed and studied [43]. It was shown in [43] under this semantics that singleton attractor detection (i.e., detection of a fixed-point) remains NP-complete but reachability and attractor membership problems can be solved better than in PSPACE (under reasonable assumptions on the complexity classes).

## 3 Detection and enumeration of singleton attractors

One of the simplest methods for detecting or enumerating attractors is to repeat the state transitions with all possible global states as initial states for a given BN and to find out which state each initial state finally reaches. This task can be done by constructing and analyzing the state transition diagram. However, the state transition diagram for a BN with *n* nodes consists of $2^{n}$ nodes and $2^{n}$ edges. Therefore, if *n* is large, it is impossible to construct and analyze the state transition diagram. In fact, the singleton attractor detection problem has been shown to be an NP-hard problem by using a reduction from 3-SAT (Boolean satisfiability problem) [6], [17]. Thus, various algorithms that work efficiently for specific classes of BNs have been proposed. In this section, we introduce singleton attractor detection/enumeration algorithms for BNs with maximum indegree *K*, BNs in which each Boolean function is AND or OR of literals, and BNs configured by nested canalyzing functions.

### 3.1 Singleton attractor detection for BNs with maximum indegree *K*

This section deals with BNs with maximum indegree *K*, where *K* is any constant larger than 1. Since the number of states of *K* input nodes is $2^{K}$ and both 0 and 1 can be assigned as an output value for each of such states, there exist $2^{2^{K}}$ Boolean functions with *K* inputs. That is, if *K* is a constant, each Boolean function is specified by a constant space and can be evaluated in constant time.

#### 3.1.1 Reduction to SAT

We have already mentioned that SAT is used to show the NP-hardness of the attractor detection problem, but it can also be applied to the singleton attractor detection itself [44], [45], [46]. More specifically, the singleton attractor detection for BNs with maximum indegree *K* can be replaced by the (K+1)-SAT of *n* variables. SAT is a problem of determining whether there is a 0-1 vector (*assignment*) a that satisfies f(a)=1 when a Boolean function *f* is given by the following *conjunctive normal form* (CNF),

$$C(x)=\underset{i}{⋀}\underset{j}{⋁}\ell_{i,j},$$

where $\ell_{i,j}$ is the *j*th literal of the *i*th clause. Note that ℓ is called a *literal* if it is a Boolean variable or its negation, and OR of literals is called a *clause*. Especially when each clause consists of at most *k* literals, it is called *k*-SAT. For example, for a BN with K=2, we consider the following transformations for the two-variable Boolean operations, ∧ (conjunction, AND), ∨ (disjunction, OR), and ⊕ (exclusive OR, XOR) (the cases of constant and unary functions are omitted since they are trivial).

$$\begin{array}{cc} x_{i}=\ell_{j}\wedge \ell_{k}\Leftrightarrow & (\overset{‾}{x_{i}}\vee \ell_{j}\vee \ell_{k})\wedge (x_{i}\vee \overset{‾}{\ell_{j}})\wedge (x_{i}\vee \overset{‾}{\ell_{k}}),\\ x_{i}=\ell_{j}\vee \ell_{k}\Leftrightarrow & (\overset{‾}{x_{i}}\vee \ell_{j})\wedge (\overset{‾}{x_{i}}\vee \ell_{k})\wedge (x_{i}\vee \overset{‾}{\ell_{j}}\vee \overset{‾}{\ell_{k}}),\\ x_{i}=\ell_{j}⊕\ell_{k}\Leftrightarrow & (\overset{‾}{x_{i}}\vee \ell_{j}\vee \ell_{k})\wedge (\overset{‾}{x_{i}}\vee \overset{‾}{\ell_{j}}\vee \overset{‾}{\ell_{k}})\wedge (x_{i}\vee \overset{‾}{\ell_{j}}\vee \ell_{k})\wedge (x_{i}\vee \ell_{j}\vee \overset{‾}{\ell_{k}})), \end{array}$$

where, in a singleton attractor, $x_{i}(t+1)=x_{i}(t)$ holds for all i=1,…,n, so *t* is omitted to express the Boolean variables. This means that each Boolean function can be converted to a 3-SAT consisting of at most four clauses. Furthermore, by applying this transformation to all Boolean functions assigned to $x_{i}$ (i=1,….,n) and then combining them with ∧, the singleton attractor detection problem of a BN with *n* nodes and K=2 can be converted into a 3-SAT problem consisting of *n* variables and at most 4n clauses. In general, the singleton attractor detection for a BN with *n* variables and the maximum indegree *K* can be converted in polynomial time to (K+1)-SAT with at most $2^{K+1}\cdot n$ clauses and *n* variables. Although readers may think it is strange to use reduction to (K+1)-SAT (not to *K*-SAT), it is reasonable because 2-SAT is solvable in polynomial time whereas singleton attractor detection for K=2 is NP-hard [6]. To actually find a singleton attractor, we need to apply an efficient SAT solver after converting to SAT by the above method. Although SAT is an NP-hard problem, various practical solvers were developed which can solve large-scale SAT instances [47]. Furthermore, various theoretically efficient algorithms have also been developed. For example, an $O(1.3303^{n})$ time algorithm is known for 3-SAT [48], which implies that the singleton attractor detection problem can be solved in $O(1.3303^{n}\cdot \mathrm{poly}(n))$ time for BNs with K=2, where poly(n) means some polynomial function of *n*.

#### 3.1.2 Using feedback vertex set

**Fig. 2:**
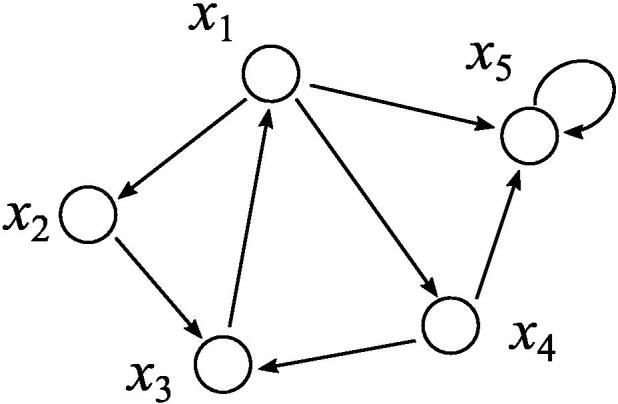
Example of a BN for illustration of FVS. $\left\{x_{1},x_{5}\right\}$ is the minimum FVS. $\left\{x_{2},x_{4},x_{5}\right\}$ is a FVS but is not the minimum one.

The singleton attractor detection can also be done by using the *feedback vertex set* (FVS) in graph theory [16]. The FVS is a subset of nodes U⊆V in a directed graph G(V,E) such that removal of all incoming edges to *U* eliminates all directed cycles (Fig. 2). In particular, the FVS with the smallest number of nodes is called the minimum FVS. If the state of each node v∈U included in FVS is fixed, the state of IN(v) is irrelevant to the update of *v*, so that, the state of FVS is propagated to all nodes except FVS in at most n-1 steps in a graph *G* with *n* nodes. In other words, by fixing the state of FVS of a directed graph G(V,E) corresponding to a given BN N(V,F), the BN reaches a stable state in at most n-1 steps. However, it should be noted that not all stable states correspond to singleton attractors. For example, if FVS = $\left\{v_{1},v_{2},v_{3}\right\}$ and $v_{1}(t+1)=v_{2}(t)\wedge v_{3}(t)$, BN reaches a specific stable state by fixing $\left[v_{1},v_{2},v_{3}]=[0,1,1\right]$, but this state is not a singleton attractor for a given BN since $v_{1}$ must be 1. Therefore, in order to enumerate singleton attractors, it is enough to assign all possible states to the minimum FVS and to update the states until the BN reaches stable states, and then to check their consistencies. Assuming that the size of minimum FVS is |U| and each Boolean function of N(V,F) can be evaluated in polynomial time, enumeration of all singleton attractors can be done in $O(2^{\left|U\right|}\cdot \mathrm{poly}(n))$ time since the number of possible states of FVS is $2^{\left|U\right|}$. It is known that the problem of finding an FVS is NP-hard, but some practical algorithms have been proposed [49], [50], [51], and they can be used for finding singleton attractors. The FVS-based algorithm has been extended for enumeration of periodic attractors in BNs [6] and for more general non-linear models of biological networks [52]. In addition, Mori and Mochizuki also reported the relationship between the expected number of singleton attractors and the feedback arc sets [53].

#### 3.1.3 Recursive algorithms

Here, we introduce a simple recursive algorithm having a guaranteed average-case complexity for singleton attractor enumeration [54]. This recursive algorithm considers a partial global state $\left[x_{1},\ldots ,x_{m}\right]$ for m<n, and iteratively examines whether there is a contradiction between the partial global states and $f_{i}(x)$ assigned to $x_{i}$ while incrementing *m*. If there is no contradiction between all $x_{i}$ and $f_{i}(x)$, output it as a singleton attractor, otherwise stop examining the current assignment and proceed to the next assignment. For example, consider the BN shown in Fig. 1(a) as an example and give x=[∊,∊,∊] as an initial state, where ∊ represents a state that has not yet been determined. Then, we examine the partial global state of x=[0,∊,∊]. At this time, $x_{2}$ and $x_{3}$ are undetermined, thus f(x) is consistent. Therefore, we expand the subset of nodes to be assigned and examine x=[0,0,∊]. Since there is no contradiction in f(x) as well, we further expand the subset and examine x=[0,0,0]. Here, f(x) is consistent and the states of all nodes are determined. Therefore, x=[0,0,0] is reported as a singleton attractor. In the next step, x=[0,0,1] is examined. Since $f_{3}(x)\;\neq \;x_{3}$, x is not a singleton attractor and nothing is output. Then, we go back to the previous recursion level and examine x=[0,1,∊]. In this case, although $x_{3}$ is not yet determined, $f_{1}(x)\;\neq \;x_{1}$ is already established, so that the examination of [0,1,∊] is stopped and then x=[1,∊,∊] is examined. By repeating this procedure, it is possible to list all singleton attractors. Letting $s=\frac{m}{n}$, theoretical analysis shows that this algorithm works in $O({\left(\max_{s}{\left(2-s^{K}\right)}^{s}\right)}^{n}\cdot \mathrm{poly}(n))$ time on the average, where the average is taken over all NK models, and 0<s<1. For example, the resulting time complexity is $O(1.35^{n})$ and $O(1.67^{n})$ for K=2 and K=10, respectively. Therefore, when *K* is small, it is sufficiently faster than $O(2^{n})$. In this algorithm, the partial global states are determined in a given order of $x_{1},\ldots ,x_{n}$. However, if the nodes are sorted in advance in the descending order of their outdegrees, the number of nodes whose states are determined at each recursive step will increase, so that it is expected that the singleton attractor can be detected with a smaller number of trials. Actually, the algorithm based on this idea has been shown to work in $O(1.19^{n})$ time and $O(1.57^{n})$ time for K=2 and K=10, respectively. Furthermore, this algorithm can be extended for enumeration of periodic attractors with a small fixed period *p* [54].

### 3.2 Singleton attractor detection for AND/OR BNs

This section considers AND/OR BNs, in which each Boolean function is AND or OR of literals. For singleton attractor detection for AND/OR BNs, there is an algorithm that works in $O(1.792^{n})$ time, which is a combination of recursive calls and existing methods for solving SAT [55]. To explain the algorithm, first we assume that the following BN function is assigned for $x_{i}$.

$$x_{i}=x_{1}\wedge x_{2}\wedge \ldots \wedge x_{h}\text{.}$$

In this case, the consistent 0-1 assignments for $\left[x_{1},x_{i}\right]$ are [0,0],[1,0], and [1,1], whereas [0,1] is inconsistent since $x_{i}$ must be 0 if $x_{1}$ is 0, so that the assignment for $\left[x_{1},x_{i}]=[0,1\right]$ does not need to be considered anymore. Thus a singleton attractor may be obtained by examining the other three assignments and repeating the same procedure for the other nodes. Since only 3 among $2^{2}=4$ assignments are examined per two nodes, this might lead to an $O(3^{n/2}\cdot \mathrm{poly}(n))=O(1.733^{n}\cdot \mathrm{poly}(n))$ time algorithm. However, this procedure cannot be continued if there are no remaining edges or only nodes with self-loops. Let *U* be the set of nodes whose 0-1 assignments are already determined by this procedure. In such case, the assignments of the set of remaining nodes *W* (i.e., W=V⧹U) are determined by the following procedure.

• if |U|>αn, examine all possible assignments on *W*,
• otherwise, compute consistent assignments on *W* using a SAT algorithm.

It is shown that by letting α=0.767 and utilizing an $O(1.234^{m}\cdot \mathrm{poly}(n))$ time algorithm for SAT with *m* clauses [56], the resulting algorithm works in $O(1.792^{n})$ time. By further omitting unnecessary examinations of partial assignments, an $O(1.587^{n})$ time algorithm was developed [57]. It is to be noted that these results might be slightly improved by using a recent $O(1.223^{m}\cdot \mathrm{poly}(n))$ time algorithm for SAT with *m* clauses [58].

### 3.3 Singleton attractor detection for nested canalyzing BNs

A Boolean function $f_{v}$ (assigned to a node *v*) is called *nested canalyzing* if it has the following form:

$$f_{v}=\ell_{1}\vee \cdots \vee \ell_{k_{1}-1}\vee (\ell_{k_{1}}\wedge \cdots \wedge \ell_{k_{2}-1}\wedge (\ell_{k_{2}}\vee \cdots \vee \ell_{k_{3}-1}\vee (\cdots ))),$$

where $\ell_{i}\in \{x_{1},\overset{‾}{x_{1}},x_{2},\overset{‾}{x_{2}},\ldots ,\overset{‾}{x_{n}}\}$ and $1⩽k_{1}<k_{2}<\cdots$. A BN is called an *nc-BN* if nested canalyzing functions are assigned to all nodes. It should be noted that both AND and OR functions of literals are special cases of nested canalyzing functions. Since biologically important functions often have a nested canalyzing form [22], [23], [24], [25], [26], it is meaningful to consider attractor detection algorithms for nc-BNs.

#### 3.3.1 Recursive algorithm for singleton attractor detection for nc-BNs

Here, we briefly introduce the basic idea of a recursive algorithm SattNC for singleton attractor detection for nc-BNs [19].

The first OR part of $f_{v}$ (i.e., $\ell_{1}\vee \cdots \vee \ell_{k_{1}-1}$) is called the *initial clause*. Suppose that *u* appears positively in the initial clause of some $f_{v}$ where u≠v. If 1 is assigned to *u*, then we have $f_{v}=1$ and thus the state of *v* becomes 1 regardless of the state of other input nodes to *v*. This means that assigning 1 to *v* determines the states of two nodes (*u* and *v*). On the other hand, if 0 is assigned to *u*, the state of *v* may not be determined. Conversely, suppose that *u* appears negatively in $f_{v}$ (i.e., $f_{v}=\overset{‾}{u}\vee f_{v}\prime$). In this case, u=0 determines the states of two nodes (*u* and *v*), whereas u=1 determines the state of one node (*u*). This procedure may be applied recursively. Let G(q) be the number of recursive calls with *q* unassigned variables in such a recursive procedure. Then, we may have

G(q)⩽G(q-1)+G(q-2)

from the above discussion. If we can repeat this procedure until the states of all nodes are determined, G(q) will be $O(1.619^{q})$ (the Fibonnaci number). However, in many cases, this procedure cannot be repeated so many times. Furthermore, assigning u=1 (resp., u=0) gives a constraint $f_{u}=1$ (resp., $f_{u}=0$). In order to cope with these issues, we need to solve SAT for a set of nested canalyzing functions, which is more general than the standard SAT for clauses (i.e., a set of OR of literals). SattNC was obtained by combining the above recursive procedure with a newly developed SAT algorithm for nested canalyzing functions, and was shown to work in $O(1.871^{n})$ time [19].

#### 3.3.2 Singleton attractor detection for nc-BNs with bounded treewidth

**Fig. 3:**
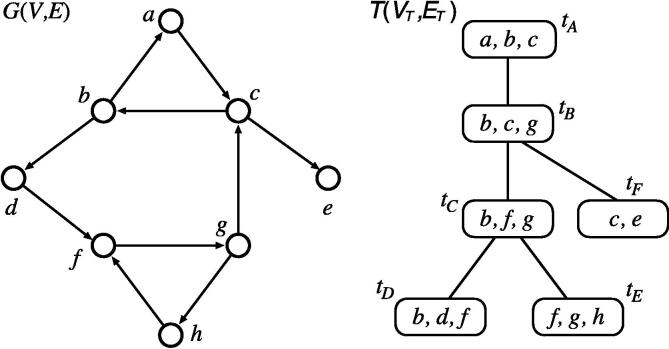
Example of graph G(V,E) and its tree decomposition $\langle T(V_{T},E_{T}),{\left(B_{t}\right)}_{t\in V_{T}}\rangle$ with width two, where $t_{A},\ldots ,t_{F}$ are bags.

It is known that many NP-hard problems can be solved in polynomial time using dynamic programming if an input graph has a fixed *treewidth*, where the treewidth is an integer value measuring how close a graph is to a tree [59], [60]. To formally define the treewidth, we consider the *tree decomposition* that transforms G(V,E) to a pair $\langle T(V_{T},E_{T}),{\left(B_{t}\right)}_{t\in V_{T}}\rangle$, where $T(V_{T},E_{T})$ is a rooted tree, ${\left(B_{t}\right)}_{t\in V_{T}}$ is a family of subsets of nodes $V=\{v_{1},\ldots ,v_{n}\}$, each $B_{t}$ is called a *bag*, each node $v_{i}\in V$ must appear in at least one $B_{t}$, nodes in each edge e∈E must be included in at least one $B_{t}$, and $B_{t}$’s containing each node must be connected in T (see Fig. 3). Note that the tree decomposition is not uniquely determined. The width of the decomposition is defined by $\max_{t\in V_{T}}(|B_{t}|-1)$and the treewidth of *G* is the smallest width among all tree decompositions of *G*.

**Fig. 4:**
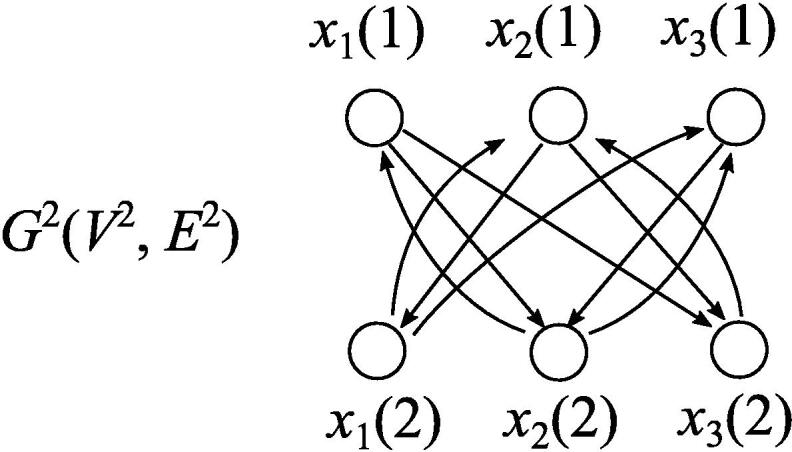
*p*-multiplied network $G^{2}=(V^{2},E^{2})$ corresponding to G(V,E) given in Fig. 1(a).

It is known that the singleton attractor detection problem can be solved in $O(n^{2(w+1)}\cdot \mathrm{poly}(n))$ if a given BN N(V,F) is composed of nested canalyzing functions and the treewidth of G(V,E) is bounded by *w* [61]. This algorithm applies dynamic programming to $T(V_{T},E_{T})$ and computes partial assignments from leaves to the root in a bottom-up manner. Chang et al. have improved this algorithm for the special cases of AND/OR BNs and nc-BNs [62]. There is also an algorithm that reduces singleton attractor detection for nc-BNs with bounded treewidth to a *constraint satisfaction problem* for bounded treewidth [61], [63].

## 4 Detection of periodic attractors

As mentioned in Section 1, detection of a periodic attractor seems much harder than detection of a singleton attractor. Nevertheless, some $o(2^{n})$ time algorithms have been developed for detection of periodic attractors with a short period. This section briefly introduces such algorithms.

### 4.1 Reduction to the singleton attractor detection

A simple strategy for detecting periodic attractors is to reduce it to the singleton attractor detection problem. Here, we introduce a simple reduction algorithm that constructs *p*-*multiplied network* $N^{p}(V^{p},F^{p})$ from an input BN N(V,F) [61]. $N^{p}(V^{p},F^{p})$ is defined as follows:

$$\begin{array}{cc} V^{p}= & \{x_{i}^{\left(1\right)},x_{i}^{\left(2\right)},\cdots ,x_{i}^{\left(p\right)}|x_{i}\in V\},\\ F^{p}= & \{f_{i}^{\left(1\right)}(x_{i_{1}}^{\left(p\right)},\cdots ,x_{i_{k_{i}}}^{\left(p\right)}),\\ & f_{i}^{\left(2\right)}(x_{i_{1}}^{\left(1\right)},\cdots ,x_{i_{k_{i}}}^{\left(1\right)}),\cdots ,\\ & f_{i}^{\left(p\right)}(x_{i_{1}}^{\left(p-1\right)},\cdots ,x_{i_{k_{i}}}^{\left(p-1\right)})\\ & |f_{i}(x_{i_{1}},\ldots ,x_{i_{k_{i}}})\in F\mathrm{and}f_{i(t)}≅f_{i}\}, \end{array}$$

where each $x_{i}^{\left(t\right)}$ is regarded as a node and $f_{i}^{\left(t\right)}≅f_{i}$ means that $f_{i}^{\left(t\right)}$ is the same Boolean function as $f_{i}$ except that input variables are different (see Fig. 4). A singleton attractor at $N^{p}(V^{p},F^{p})$ clearly corresponds to an attractor of N(V,F) with period *q* that divides *p*. However, in order to guarantee that the detected attractor has exactly period *p*, for all q=2,…,p, $x_{i}^{\left(1\right)}\;\neq \;x_{i}^{\left(q\right)}$ must hold for some $x_{i}\in V$. This means that the state of N(V,F) at time t=1 must be different from that of at time t=2,…,p, so that N(V,F) does not take the same global state at $t=q_{1}$ and $t=q_{2}$ $\left(1⩽q_{1}<q_{2}⩽p\right)$. Here, let ϕ(t) be a function from {2,…,p} to {1,…,n} and ψ(t) be a function from {2,…,p} to {0,1}. To check if there is a periodic attractor with period *p* of N(V,F), it is enough to check whether there exist an singleton attractor of $N^{p}(V^{p},F^{p})$ and functions ϕ(t) and ψ(t) such that $x_{\phi (t)}^{\left(1\right)}=\psi (t)$ and $x_{\phi (t)}^{\left(t\right)}=1-\psi (t)$ holds for all t=2,…,p. The possible number of ϕs and ψs are $n^{p-1}$ and $2^{p-1}$, respectively, which are in polynomial of *n* for a constant *p*. Therefore, if singleton attractor detection can be done in $O({\left(1+\delta \right)}^{n})$ time, *p*-periodic attractor detection canbe done in $O({\left(1+\delta \right)}^{\mathrm{pn}}\cdot \mathrm{poly}(n))$ time. However, even if the $O(1.587^{n})$ time algorithm for singleton attractor detection for AND/OR BNs is applied, it does not yield an $o(2^{n})$ time algorithm because it would take $O(1.587^{2n}\cdot \mathrm{poly}(n))$ time ($1.587^{2}\approx 2.519$) to detect a periodic attractor with period 2.

### 4.2 2-periodic attractor detection for AND/OR BNs

This section describes a 2-periodic attractor detection algorithm for AND/OR BNs using $N^{2}(V^{2},F^{2})$ [61]. First, we transform an AND/OR BN to an OR BN. Let $x_{i}$ be an AND node assigned the following function:

$$x_{i}(t+1)=\ell_{i_{1}}\wedge \ldots \wedge \ell_{i_{k}}\text{.}$$

This Boolean function can be transformed to an OR function by replacing it to

$$x_{i}(t+1)=\overset{‾}{\ell_{i_{1}}}\vee \ldots \vee \overset{‾}{\ell_{i_{k}}}$$

and negating all $x_{i}(t+1)$ in the function $f_{j}$ for all $x_{j}\in V$. Therefore, in this section, we can assume that $N^{2}(V^{2},F^{2})$ is an OR BN.

The basic strategy of the 2-periodic attractor detection algorithm is similar to that of the $O(1.587^{n})$ time algorithm for an AND/OR BN, that is, we recursively examine 0-1 assignments and finally apply an SAT algorithm. However, here we use the special property on $N^{2}(V^{2},F^{2})$. The procedure of 2-periodic attractor detection is to first construct $N^{2}(V^{2},F^{2})$ and set all nodes to be unassigned. Then, we recursively examine 0-1 assignments for unassigned nodes *x* such that U(x)⩾3 until no such nodes exist or the number of assigned nodes is more than *H*, where U(x) is the number of unassigned neighboring nodes of *x* and *H* is a parameter. Here, let *A* be the set of assigned nodes, and let $A_{1}=A\cap V_{1}^{2}$ and $A_{2}=A\cap V_{2}^{2}$ (w.l.o.g., $\left|A_{1}|⩾|A_{2}\right|$), where $V_{1}^{2}$ and $V_{2}^{2}$ are the sets of nodes $V^{2}$ corresponding to t=1 and t=2, respectively. If |A|>H, we examine all possible assignments for $V_{1}⧹A_{1}$, otherwise recursively examine assignments for paths and cycles (because all nodes with U(x)⩾3 have already been assigned), and then finally solve SAT. The resulting algorithm has been shown to work in $O(1.985^{n})$ time by letting H=0.3196n [61]. It is known that detection of 2-periodic attractor can be done in linear time for a positive OR BN in which all Boolean functions are OR functions and all variables appear positively [21], [61]. However, periodic attractor detection remains NP-hard for a positive BN in which each function is an AND or OR function [64].

### 4.3 Periodic attractor detection for nc-BNs with bounded treewidth

The algorithm presented in Section 3.3.2 can be extended for detection of a *p*-periodic attractor for an nc-BN with bounded treewidth, using the *p*-multiplied network $N^{p}(V^{p},F^{p})$ [61]. The extended algorithm is based on the following proposition: if the graph G(V,E) associated with N(V,F) has the treewidth *w*, the graph $G^{p}(V^{p},F^{p})$ associated with $N^{p}(V^{p},F^{p})$ has a tree decomposition with the width less than p(w+1), and for each x∈V, $x^{\left(1\right)},\ldots ,x^{\left(p\right)}$ are included in the same $B_{t}$ [61]. Then, we can apply a dynamic programming procedure to the tree decomposition of $G^{p}(V^{p},F^{p})$ associated with $N^{p}(V^{p},F^{p})$, where some modifications are needed to detect an attractor with exactly period *p*. The resulting algorithm works in $O(n^{2p(w+1)}\cdot \mathrm{poly}(n))$ time [61]. Furthermore, the algorithm works in O(g(d,p,w)·poly(n)) time if the maximum indegree is bounded by a constant *d* where *g* is a non-polynomial function depending on d,p,w but not depending on *n*. It gives a fixed-parameter algorithm when p,w, and *d* are parameters (i.e., the exponential factor of the time complexity depends only on some parameters for the algorithm) [59], [60].

## 5 Practical algorithms

In the previous sections, we have introduced singleton/periodic attractor detection and enumeration algorithms that are guaranteed to work in less than $O(2^{n})$ time. However, these algorithms are limited to particular subclasses of BNs and no such an algorithm has been known for general BNs. On the other hand, there are many algorithms that do not have such a theoretical guarantee but work efficiently in practice.

### 5.1 SAT and logic-based approaches

One major practical approach is to use SAT and related logic-based methods. As mentioned in Section 3.1.1, attractor detection problems can be reduced to SAT. Furthermore, many practically efficient SAT solvers have been developed [44], [45]. Therefore, it is reasonable to develop practically efficient attractor detection/enumeration methods for BNs and related models using such SAT solvers. For example, Dubrova and Teslenko used a SAT solver to search for a path of length *p* on the state transition diagram and detected an attractor by checking if it contains a loop [45], and de Jong and Page transformed the problem of searching for a stable state of a network described by the pairwise-linear differentiation equation model into SAT [44]. For other logic-based approaches, Devloo et al. developed an algorithm applying constraint programming to the singleton attractor detection and enumeration [65]. Inoue provided an algorithm that directly encodes a BN into a logic program and computes a singleton attractor based on that logic program [66], and Abdallah et al. proposed an algorithm based on *answer set programming* (ASP) that enumerates all attractors without creating an entire state transition diagram [67]. In addition to SAT, *Binary decision diagrams* (BDDs) have also been utilized to solve large scale logic-based problems in various fields. Therefore, it is reasonable to apply BDDs in place of SAT solvers. Indeed, some methods have been developed based on BDDs that can detect attractors for large networks [68], [69], [70]. Integer linear programming (ILP) is another useful method to efficiently solve Boolean constraints and thus has been applied to attractor detection and related problems on BNs [71], [72], [73].

### 5.2 Network reduction-based approaches

Another major practical approach is to use network reduction. For example, suppose that *v* has an input node *u* and *u* has only one input node *w*. Then, for both detection and enumeration of singleton attractors, we can remove *u* by letting *w* as an input to *v*. Although this example is a very simple one, various reduction methods were developed where logic-based or other methods were used to solve reduced instances at the final stage. For example, Veliz-Cuba et al. proposed various reduction rules including the above mentioned one, elimination of redundant edges, replacement of functions, and simplification of Boolean functions [74]. Veliz-Cuba et al. developed a singleton attractor enumeration algorithm that transforms input networks to AND-NOT networks, then applies network reduction, and finally applies an algebraic method (compute the Gröbner basis to perform a generalized version of Gaussian elimination) [75]. He et al. developed a reduction method based on removal of unstable partial states and identification of constant nodes [76]. Saadatpour et al. provided rigorous proofs of conservation of attractors in synchronous and asynchronous BNs for various reduction methods [77]. Beneš et al. proposed another network reduction method called *interleaved transition guided reduction* (ITGR) for detecting of bottom SCCs of asynchronous BNs [78]. They succeeded in handling large Boolean networks of real data (up to 350 variables) as well as of synthetic data (up to 1100 variables) based on the reduction technique. Gao et al. characterized periodic attractors of a conjunctive BN (i.e., each Boolean function is AND of literals) whose underlying directed graph is strongly connected by establishing bijection between the set of periodic attractors and the set of binary necklaces (i.e., character strings over the binary set {0,1}, where their all rotations are dealt with as equivalent) of a certain length [79]. Chen et al. extended this study to BNs over weakly connected directed graph by applying network reduction [80]. It is to be noted that network reduction methods were utilized also in some other methods mentioned in this section.

### 5.3 Network decomposition-based approaches

Divide-and-conquer is one of the major general techniques to efficiently solve various combinatorial problems in computer science. Therefore, it is reasonable to apply divide-and-conquer to the attractor detection/enumeration problems on BNs. Indeed, various methods have been developed for the problem by decomposing the original network into subnetworks and then reconstructing global attractors by integrating local attractors for the subnetworks, where the techniques introduced in Sections 5.1, 5.2 are also utilized in many of them. As a pioneering work in this direction, Irons proposed an algorithm that combines partial state and predecessors [81]. Mizera et al. developed another algorithm by using decomposition of the original BN into *strongly-connected components* (SCCs) [41], where an SCC is a well-known concept in graph theory and is a maximal subnetwork in which all node pairs are connected by directed paths. Su et al. significantly improved this algorithm by further partitioning SCCs [82]. Zañudo and Albert introduced the concept of a *stable motif*, which is an SCC that stabilizes in attractors and made use of stable motifs to efficiently find attractors in the whole BN [83]. Klarner et al. introduced a similar concept, *trap space*, and combined it with ILP [72] and model checking [84]. Choo and Cho developed a method based on hierarchically partitioning with focusing on attractors corresponding to a particular phenotype of interest instead of considering all attractors [85]. Tamaki developed an algorithm combining *path-decomposition* and partial states [86]. It should be noted that the *tree decomposition*-based methods mentioned in Sections 3.3.2, 4.3 can also be regarded as network decomposition-based ones.

### 5.4 FVS-based approaches

The FVS-based singleton attractor detections for synchronous BNs are described in Section 3.1.2. Meanwhile, practical FVS-based approaches for asynchronous random Boolean networks (ARBNs) have also been proposed [42], [87]. Skodawessely and Klemm focused on a *reduced dynamics* to make it easier to find attractors of ARBNs [87]. A reduced dynamics is obtained by retaining a Boolean state of a node in FVS and then removing state transitions from the state transition diagram. Finally, the attractors of an ARBN can be found from the sets of fixed points of the reduced dynamics. Although this approach seems to be widely applicable, it is often difficult to handle large networks (n⩾30) since it requires large memory space to store all the state vectors of an attractor during the traverse of the original state transition diagram. Van Giang et al. were inspired by the concept of reduced dynamics and presented the relations between FVSs and dynamics of BNs with formal proofs [42]. Furthermore, they proposed another FVS-based method for detecting attractors in ARBNs based on the relations. Its main idea is to systematically remove edges from the transition diagram and then filter out the set of fixed points of them to get a candidate set of states which one-to-one corresponds to the set of attractors of the given ARBN. The computational experiments using real biological networks and random *N*-*K* networks showed that the method succeeded in handling large networks whose sizes are up to 101 without any network reductions.

### 5.5 Other approaches

It is well-known that starting from an arbitrary state and repeating update of states, the trajectory will finally fall into a singleton or periodic attractor. Therefore, starting from many initial states, we may be able to get directed attractors or some statistics on attractor distributions. Although such sampling-based studies have been conducted on small-size BNs, it is difficult to apply such methods to analysis of large-scale BNs.

Another major approach is to use *semi-tensor product* (STP) [5], which is an extension of matrix product. In this approach, many problems on BNs are defined as matrix-based problems. Since matrices play central roles in control theory, various concepts and methodologies in control theory have been applied to BNs and thus many studies have been done using STP [88]. For example, a state transition diagram can be represented as a binary matrix *A* of size $2^{n}\times 2^{n}$ in which $A_{\mathrm{ij}}=1$if and only if there exists a transition from state *i* to state *j* [5]. Then, singleton attractors correspond to elements of *A* such that $A_{\mathrm{ii}}=1$. Therefore, once *A* is constructed, detection and enumeration of singleton attractors become trivial [5]. Although most of existing STP-based methods need to handle $2^{n}\times 2^{n}$ or larger size matrices and thus can only handle small-size BNs, some efforts have been done to address this complexity issue [89], [90].

## 6 Conclusion

In this article, we briefly reviewed algorithms for singleton/periodic attractor detection/enumeration with focusing on synchronous BNs. Enumeration of all singleton and periodic attractors can trivially be done by constructing and analyzing a state transition diagram. However, such an approach needs $O(2^{n}\cdot \mathrm{poly}(n))$ or more computation time because there exist $2^{n}$ possible states. Since $2^{n}$ is often too large, it is important to develop theoretically and/or practically efficient algorithms. However, it is not an easy task because it is known that detection of a singleton attractor is NP-hard, enumeration of a singleton attractor is #P-hard, and detection of a long periodic attractor is suggested to be PSPACE-hard [6].

We have seen that singleton attractor detection can be done in provably faster than in $O(2^{n})$ time (i.e., still exponential, but $o(2^{n})$ time) for some special but reasonably wide classes of BNs such as AND-OR BNs and BNs consisting of nested canalyzing functions, by combining recursive assignment techniques with SAT algorithms. However, these presented algorithms are not necessarily optimal. Therefore, improvement of the time complexity of these algorithms is left as an open problem. We have also seen that singleton attractor detection can be done in polynomial time for BNs with bounded treewidth and bounded maximum degree, using tree decomposition and dynamic programming as in many other polynomial-time algorithms for graphs with bounded treewidth. As for enumeration of singleton attractors, we have reviewed a simple recursive algorithm that works in $o(2^{n})$ time in the average case.

Detection of a periodic attractor is much more difficult. We have seen that detection of an attractor with period two can be done in $o(2^{n})$ time for AND-OR BNs. However, as far as we know, there is no $o(2^{n})$ time algorithm that can detect an attractor with period three (or more) for reasonably wide classes of BNs. Therefore, development of such an algorithm is left as an open problem. Recently, in order to cope with this difficulty, an algorithm has been proposed which detects a long periodic attractor in $o(2^{n})$ expected time, assuming that partial 0-1 assignments in a desired attractor is probabilistically given in advance as *a priori* [15]*. This method succeeded in detecting attractors of large networks with long periods that were difficult to find by existing methods.*

From a practical viewpoint, many methods have been developed for detection/enumeration of singleton/periodic attractors for synchronous and asynchronous BNs. However, it is unclear which methods are the most useful in practice, where it may depend on structures of the target BNs. Therefore, rigorous comparison of state-of-the-art methods should be done. Furthermore, it seems that detection/enumeration of long attractors remains quite difficult also in practice. Therefore, practically efficient methods should be developed for detection/enumeration of long attractors.

## CRediT authorship contribution statement

**Tomoya Mori:** Investigation, Writing – original draft. **Tatsuya Akutsu:** Conceptualization, Investigation, Writing – original draft.

## Declaration of Competing Interest

The authors declare that they have no known competing financial interests or personal relationships that could have appeared to influence the work reported in this paper.
